# Differential Regulatory Effects of Probiotics on Bone Metabolism by the Status of Bone Health and Delivery Route

**DOI:** 10.1007/s12602-024-10441-x

**Published:** 2024-12-28

**Authors:** Chaeyeon Park, Ok-Jin Park, Yeongkag Kwon, Jueun Lee, Cheol-Heui Yun, Seung Hyun Han

**Affiliations:** 1 Department of Oral Microbiology and Immunology, and Dental Research Institute, School of Dentistry, Seoul National University, Seoul, Republic of Korea; 2 Department of Agricultural Biotechnology, and Research Institute of Agriculture and Life Sciences, Seoul National University, Seoul, Republic of Korea

**Keywords:** Probiotics, *Lactiplantibacillus plantarum*, Bone metabolism, Delivery route

## Abstract

Probiotics are known to have favorable effects on human health. Nevertheless, probiotics are not always beneficial and can cause unintended adverse effects such as bacteremia and/or inflammation in immunocompromised patients. In the present study, we investigated the effects of probiotics on the regulation of bone metabolism under different health conditions and delivery routes. Intragastric administration of *Lactiplantibacillus plantarum* to ovariectomized mouse models for mimicking post-menopausal osteoporosis in humans substantially ameliorated osteoporosis by increasing bone and mineral density. In contrast, such effects did not occur in normal healthy mice under the same condition. Interestingly, however, intraperitoneal administration of *L. plantarum* induced bone destruction by increasing osteoclast differentiation and decreasing osteoblast differentiation. Furthermore, when *L. plantarum* was implanted into mouse calvarial bone, it potently augmented bone resorption. Concordantly, *L. plantarum* upregulated osteoclastogenesis and downregulated osteoblastogenesis in in vitro experiments. These results suggest that *L. plantarum* can have distinct roles in the regulation of bone metabolism depending on bone health and the delivery route.

## Introduction

Bone is a dynamic organ that undergoes continuous regeneration by the harmonized actions of osteoclasts and osteoblasts [1]. Osteoclasts are bone-resorbing cells differentiated by receptor activator of NF-κB ligand (RANKL) from monocyte/macrophage lineage of hematopoietic stem cells. As osteoclasts differentiate, they gradually express osteoclast-specific proteins such as tartrate-resistant acid phosphatase (TRAP), destroying the old bone to make new [2]. On the other hand, osteoblasts are derived from mesenchymal stem cells and play an important role in bone formation [3]. Osteoblast differentiation is mediated by a representative transcription factor, runt-related transcription factor 2 (Runx2), that induces mineralization and production of osteogenic proteins such as alkaline phosphatase (ALP) [3]. The homeostatic regulation of osteoclasts and osteoblasts is crucial for maintaining bone health. Osteoporosis can occur if homeostasis is disrupted and osteoclast activity overwhelms osteoblast activity by factors such as aging, hormonal change, and bacterial infection [4, 5].

Probiotics have the potential to serve as a therapeutic option for osteoporosis patients [6]. Microorganisms that generally offer health benefits to the host are classified into probiotics [7, 8]. Probiotics such as *Lactobacillus*, *Bifidobacterium*, and *Streptococcus* species are considered effective regulators of intestinal flora to improve host health [8–10]. They produce various beneficial bacterial products, inhibit the colonization of pathogenic bacteria, and help the host construct a healthy mucosal layer [11]. Among them, *Lactiplantibacillus plantarum* is one of the well-known probiotics with diverse health-beneficial effects. For example, *L. plantarum* can balance intestinal microbiota, enhance the function of the gastrointestinal barrier, and improve immune responses [12]. Moreover, several studies demonstrated that *L. plantarum* contributes to bone health [13, 14]. Myeong et al. reported that oral gavage of *L. plantarum* into ovariectomy (OVX)-induced osteoporotic mouse models upregulates the femoral bone mass [15]. Concordantly, the bone loss caused by OVX surgery is attenuated by oral administration of *L. plantarum* into osteoporotic rats [16, 17].

Despite the benefits of probiotic use, in recent years, some undesirable features of probiotics have emerged. Probiotic effects are not always positive and may be dependent on the bacterial strain. Also, the possibilities of horizontal antibiotic-resistant gene transfer from or to pathogens and difficulties in the maintenance of constant cell viability are relevant challenges [18]. Probiotics can cause diseases in immunocompromised patients, individuals with abnormal gastrointestinal barriers, or people undergoing recent surgical treatments [19, 20]. Indeed, it was demonstrated that probiotic supplementation induces systemic infections such as bacteremia, infective endocarditis, and pneumonia in immunocompromised patients [21, 22]. Probiotics even aggravate metabolic activities and result in excessive immune stimulation [21]. The route of drug administration is known to influence therapeutic efficacy [23]. Thus, the effects of probiotics may vary depending on the health status of consumers and the delivery route. However, no research has yet examined both the health status and the delivery routes for probiotics. *L. plantarum* was chosen for this study due to its established benefits for bone health. We investigated the different effects of *L. plantarum* on bone metabolism when administered via intragastric, peritoneal, and calvarial routes in both normal and osteoporotic mouse models.

## Materials and Methods

### Reagents and Chemicals

Recombinant murine macrophage-colony stimulating factor (M-CSF) and RANKL were purchased from JW CreaGene (Seongnam, Republic of Korea) and R&D Systems (Minneapolis, MN, USA), respectively. Fetal bovine serum (FBS) and alpha-minimum essential medium (α-MEM) were purchased from Gibco (Paisley, UK). Penicillin/streptomycin and trypsin-ethylene-diamine-tetraacetic acid (EDTA) were obtained from Hyclone (Logan, UT, USA). Each TRAP staining kit for osteoclasts and ALP detection kit for osteoblasts was obtained from Cosmobio (Tokyo, Japan) and Merck (Darmstadt, Germany), respectively. Alizarin red S staining solution, ascorbic acid, and β-glycerophosphate were obtained from Sigma-Aldrich (St. Louis, MO, USA). Antibodies specific to Runx2 and Hoechst 33258 were obtained from Cell Signaling Technology (Beverly, MA, USA) and Invitrogen (Carlsbad, CA, USA), respectively. Goat anti-rabbit IgG Alexa fluor 568 was purchased from Abcam (Cambridge, UK). De Man, Rogosa and Sharpe (MRS) was purchased from BD Biosciences (San Diego, CA, USA).

### Preparation of Heat-Treated *L. plantarum*

*L. plantarum* KCTC 10887BP (Korean Collection for Type Culture; Daejeon, Korea) is a species of Gram-positive lactic acid bacteria commonly found in fermented foods such as Kimchi. Lipoteichoic acid, a major cell wall component, of *L. plantarum* KCTC 10887BP has the anti-inflammatory effects and inhibits the biofilm formation of pathogenic bacteria [24, 25]. The bacteria were grown in MRS at 37°C to mid-log phase. It was washed with phosphate-buffered saline (PBS) twice and inactivated at 70°C for 2 h. To confirm complete inactivation of *L. plantarum*, it was plated on MRS agar plates and cultured at 37°C for more than 24 h. No bacterial colony was observed (data not shown).

### Mice

Six-week-old male C57BL/6 mice and 12-week-old female C57BL/6 mice were obtained from DooYeol Biotech (Seoul, Republic of Korea). All animal experiments were approved by the Institutional Animal Care and Use Committee of Seoul National University (Approval No. SNU-210106–1–2). We used four experimental groups. First, 12-week-old female C57BL/6 mice were bilaterally ovariectomized to create an osteoporotic mouse model. The same surgical procedure was conducted on sham control mice without ovary removal. After three weeks, we administered 2 × 10^9^ colony forming unit (CFU) of *L. plantarum* to the mice three times per week for four weeks. Second, we administered 2 × 10^9^ CFU of *L. plantarum* to six-week-old male C57BL/6 mice three times a week for four weeks to confirm the effect of *L. plantarum* on normal mice. Third, we intraperitoneally administered 2 × 10^9^ CFU of *L. plantarum* to six-week-old male C57BL/6 mice twice a week for four weeks. Fourth, a collagen sheet soaked with 1 × 10^9^ CFU of *L. plantarum* was implanted on mouse calvaria. After seven days, the collagen-implanted mice were sacrificed and the calvarial bones were obtained. 

### Micro-Computed Tomography (Micro-CT)

Micro-CT is a tool for 3D imaging that allows visualization of the interior of an object without destroying the sample. When applied to mouse bone, micro-CT evaluates bone quality by quantifying bone volume, trabecular thickness, trabecular separation, and trabecular number, while also providing detailed images of bone structure. The femur or calvaria was obtained and scanned with micro-CT (Skyscan 1272, Bruker, Kontich, Belgium). The trabecular bone parameters were calculated by CT analyzer software (Skyscan; Bruker, Kontich, Belgium). The cortical thickness of femoral bones or the resorbed area of calvarial bones was analyzed by ImageJ software (National Institutes of Health, Bethesda, MD, USA).

### Histology, Hematoxylin and Eosin (H&E) Staining, TRAP Staining, and Immunohistochemistry

After micro-CT analysis, the calvarial bones were subjected to the staining of TRAP which is secreted from the ruffled border of osteoclasts, to determine the extent of osteoclastogenesis. The femoral bones were decalcified with 10% EDTA in PBS for seven days at 4℃. Decalcified bones were embedded in paraffin, sectioned at 5 µm, and attached to glass slides. The paraffin-sectioned samples were stained for H&E or TRAP. In addition, the femur sections were stained immunohistochemically against Runx2 with anti-Runx2 monoclonal antibody and imaged with a digital upright fluorescence microscope (Olympus BX51, Olympus, Tokyo, Japan). Osteoclast surface (OC.S)/bone surface (BS) and Runx2 relative area/BS were measured by OsteoMeasure software (Osteometrics, Decatur, GA, USA) and ZEN software (Carl Zeiss, Oberkochen, Germany), respectively.

### Differentiation of Osteoclasts

Bone marrow cells were isolated from the femurs and tibiae of a mouse and incubated in α-MEM supplemented with 10% FBS, 100 U/ml penicillin, and 100 µg/ml streptomycin in the presence of 5 ng/ml of M-CSF for 24 h. Non-adherent cells were obtained and further differentiated for 4 days into bone marrow-derived macrophages (BMMs) with 30 ng/ml of M-CSF. BMMs were seeded onto a 96-well cell culture plate at 2.5 × 10^4^ cells/200 µl/well and incubated with 30 ng/ml M-CSF and 20 ng/ml RANKL for 2 days to differentiate them into committed osteoclasts. The cells were treated with heat-treated *L. plantarum* in the presence of 30 ng/ml M-CSF for 24 h. After the incubation, the mature osteoclasts were fixed in solution (8% formaldehyde, 26% citrate, and 66% acetone) and stained with a TRAP staining kit. The TRAP-stained cells with three or more nuclei were enumerated by microscopic analysis. The images of stained cells were obtained using a microscope (Olympus CKX41).

### Differentiation of Osteoblasts

MC3T3-E1 Subclone 4 (ATCC CRL-2593) cells were purchased from the American Type Culture Collection (Rockville, MD, USA) for osteoblast differentiation. The cells were maintained in α-MEM supplemented with 10% FBS, 100 U/mL penicillin, and 100 µg/mL streptomycin. The MC3T3-E1 Subclone 4 cells were seeded onto a 48-well culture plate at 2.5 × 10^4^ cells/400 µl/well and incubated with 2 mM β-glycerophosphate and 50 µg/ml ascorbic acid in the presence or absence of heat-treated *L. plantarum* for six days (ALP staining) or 12 days (alizarin red S staining). Then, the mature osteoblasts were fixed with 4% paraformaldehyde and rinsed with Tris-buffered saline buffer containing 0.05% Tween-20. The cells were stained with an ALP detection kit or alizarin red S staining solution. The images of stained cells were obtained using a microscope (Olympus CKX41). The magnitude of mineralization was quantified by dissolving calcium deposits in the cell culture with a dissolving solution (20% methanol, 10% acetic acid, and 70% distilled water).

### Statistical Analysis

All data are shown as mean ± standard deviation from more than three replicates unless otherwise stated. Treatment groups were compared with an appropriate control group, and statistical analysis was determined using Student’s *t*-test. Statistical significance (*) was determined at *P* < 0.05.

## Results

### Intragastric Administration of *L.plantarum* Restored the Femoral Bone Mass of Osteoporotic Mice

**Fig. 1:**
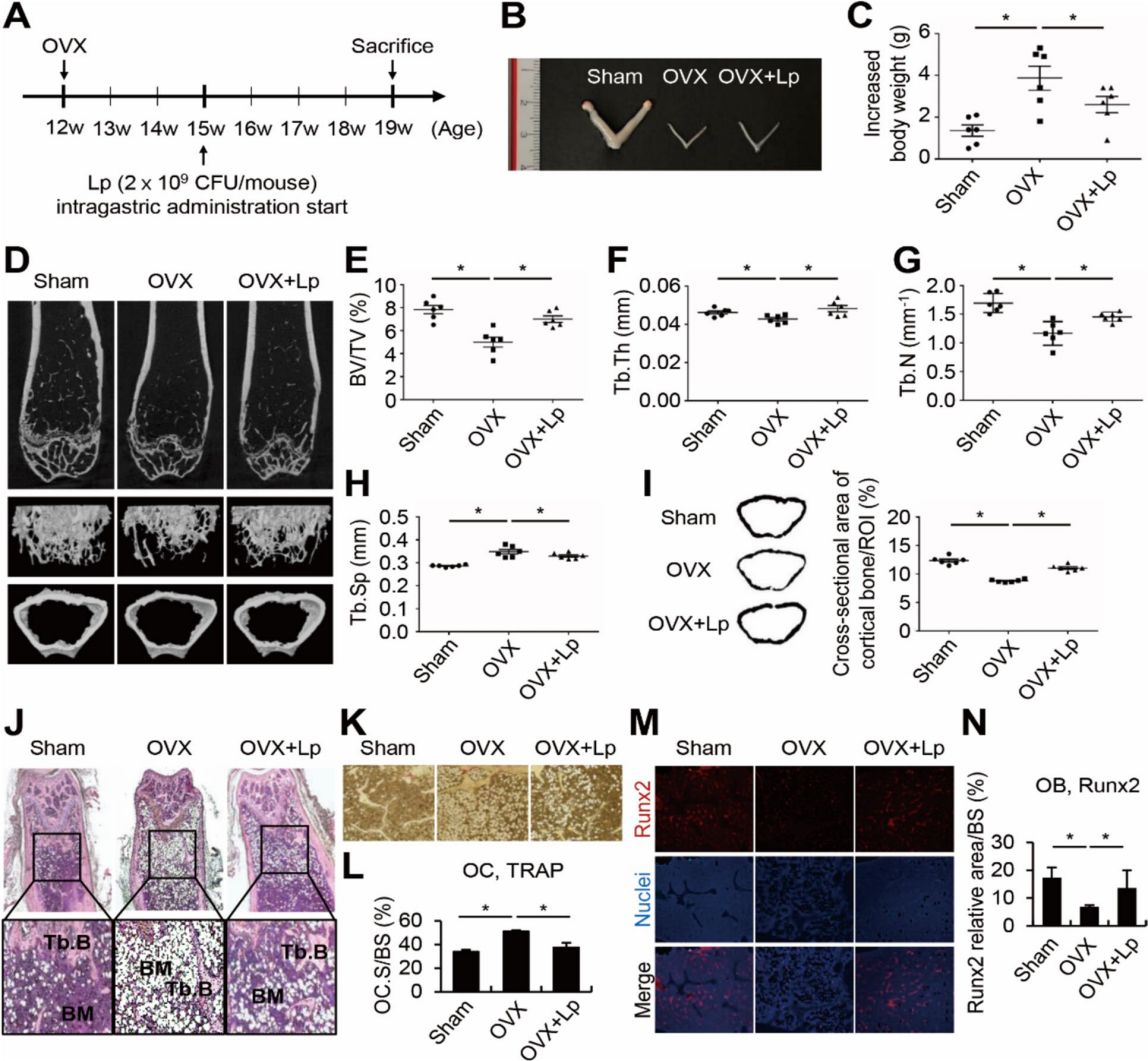
Intragastric administration of *L. plantarum* increased the bone mass of osteoporotic mice. (**A**) Twelve-week-old female C57BL/6 mice (*n* = 6) were ovariectomized. After three weeks, heat-treated *L. plantarum* (2 × 10^9^ CFU) was administered to the mice intragastrically for four weeks at a two-day interval. (**B**) At the end of the administration, mice were sacrificed, and the uterus was dissected and photographed. (**C**) The body weight of each mouse was measured at weeks 12 and 19 and the change was monitered. (**D**) The femur was scanned by micro-CT and a representative image is shown. (E-I) Bone parameters of (**E**) BV/TV, (**F**) Tb.Th, (**G**) Tb.N, (**H**) Tb.Sp, and (**I**) cortical bone thickness were calculated from 3D reconstruction of micro-CT images using a CT analyzer and ImageJ software. (J-N) Sections from the femur were stained for (**J**) H&E, (**K**, **L**) TRAP, and (**M**, **N**) Runx2. Lp, heat-treated *L. plantarum*; BV/TV, trabecular bone volume; Tb.Th, trabecular thickness; Tb.N, trabecular number; Tb.Sp, trabecular separation; ROI, region of interest; Tb.B, trabecular bone; BM, bone marrow; OC, osteoclast; OC.S/BS, osteoclast surface/bone surface; OB, osteoblast; BS, bone surface. *P < 0.05

To assess the potential benefits of intragastric administration of *L. plantarum* on osteoporotic bone regeneration, we intragastrically administered heat-treated *L. plantarum* to an OVX-induced osteoporotic mouse model mimicking post-menopausal osteoporosis in humans and measured bone parameters. Of note, heat-treated *L. plantarum* was administered in all the following experiments to exclude the possibility of active bacterial proliferation and focus on the effect of probiotic components. OVX surgery was performed on 12-week-old female mice. Three weeks after the surgery, *L. plantarum* was intragastrically administered into the OVX mice for four weeks (Fig. 1A). We confirmed that each ovary was successfully removed by OVX surgery (Fig. 1B). The body weight was increased in the OVX group compared to the sham control group, which is a typical feature of OVX mice [26] (Fig. 1C). We then analyzed the femurs by micro-CT to measure the femoral bone density of mice. *L. plantarum* upregulated femoral bone mass (Fig. 1D), which was verified by confirming that trabecular bone volume, trabecular thickness, and trabecular number were all reduced in the PBS-treated OVX mice while *L. plantarum* treatment recovered all of these parameters (Fig. 1E-G). Trabecular separation was increased in the PBS-administered OVX mice and decreased in the *L. plantarum*-treated OVX mice (Fig. 1H). In addition, the thickness of cortical bone was also augmented in the *L. plantarum*-treated OVX mice compared to the PBS-treated OVX mice, demonstrating that both trabecular and cortical bone of femur were strengthened by intragastric administration of *L. plantarum* (Fig. 1I). When the genuine cross-section of the femoral bone was confirmed through H&E staining, both trabecular and cortical bone volume was higher in the *L. plantarum*-treated OVX mice than in the PBS-treated OVX mice in micro-CT results (Fig. 1J). When comparing the *L. plantarum*-treated OVX mice to the PBS-treated OVX mice, we observed a decrease in mature osteoclasts on the bone surface (Fig. 1K and L). Conversely, there was an increase in mature osteoblasts (Fig. 1M and N). These results indicate that intragastric administration of *L. plantarum* to OVX-induced osteoporotic mice restores the bone loss caused by OVX by increasing osteoblast activity and decreasing osteoclast activity.

### Intragastric Administration of *L. plantarum* did not affect the Bone Mass of Normal Mice

**Fig. 2:**
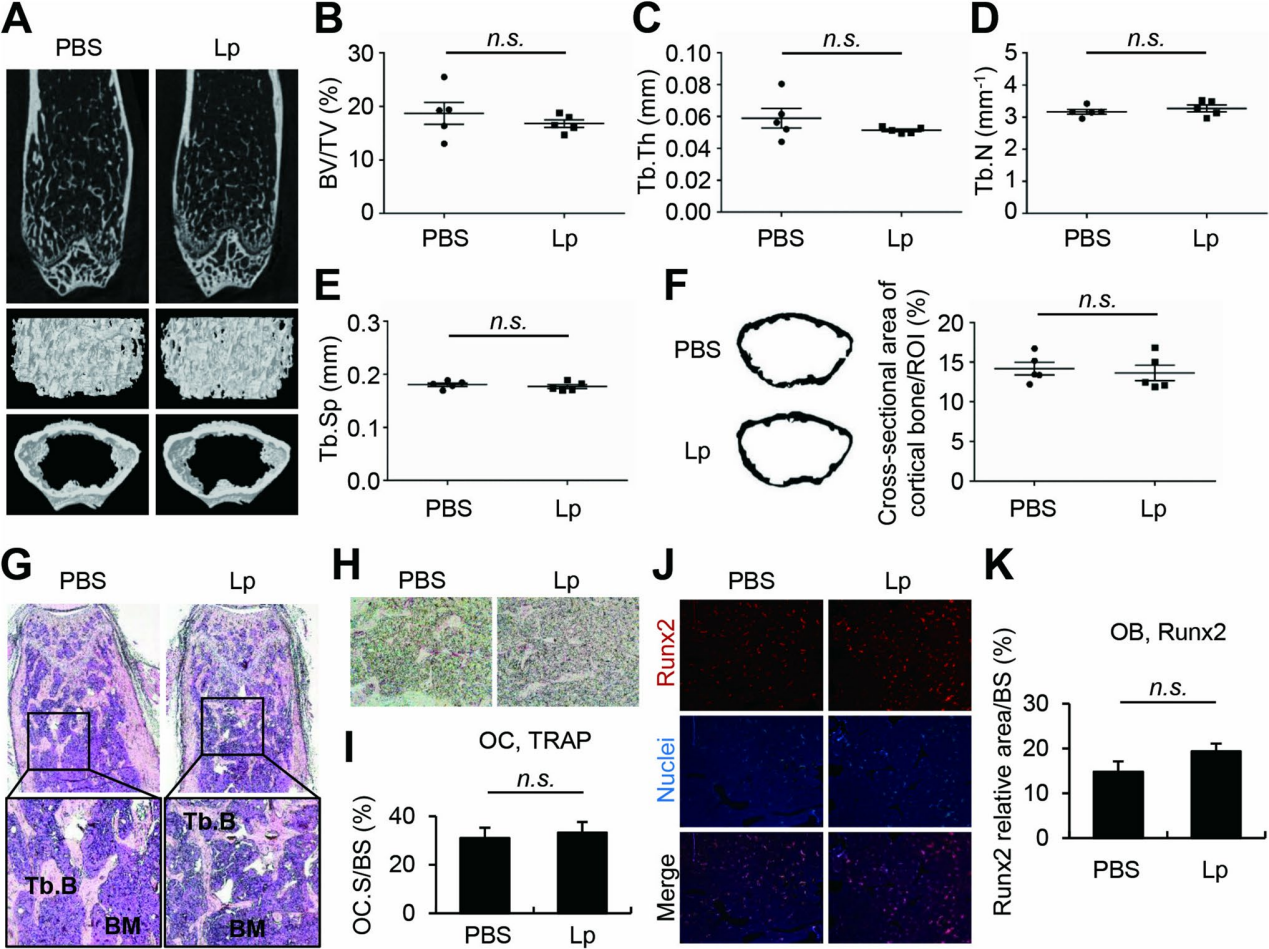
Intragastric administration of *L. plantarum* did not affect the bone mass of normal mice. C57BL/6 mice (*n* = 5) were intragastrically administered heat-treated *L. plantarum* (2 × 10^9^ CFU) for four weeks at a two-day interval. (**A**) The femurs were scanned by micro-CT and a representative image of the femur is shown. (B-F) Bone parameters of (**B**) BV/TV, (**C**) Tb.Th, (**D**) Tb.N, (**E**) Tb.Sp, and (**F**) cortical bone thickness were calculated from the 3D reconstruction of micro-CT images using a CT analyzer and ImageJ software. (G-K) Sections from the femur were stained for (**G**) H&E, (**H**, **I**) TRAP, and (**J**, **K**) Runx2. Lp, heat-treated *L. plantarum*; BV/TV, trabecular bone volume; Tb.Th, trabecular thickness; Tb.N, trabecular number; Tb.Sp, trabecular separation; ROI, region of interest; Tb.B, trabecular bone; BM, bone marrow; OC, osteoclast; OC.S/BS, osteoclast surface/bone surface; OB, osteoblast; BS, bone surface; *n.s.*, non-significant

Next, we investigated whether *L. plantarum* provides benefits to healthy normal bones as in osteoporotic bones. Six-week-old healthy normal mice were intragastrically administered *L. plantarum* for four weeks at a two-day interval and femurs were subjected to micro-CT analysis. No discernible difference was observed in images of femoral bones between mice treated with PBS and those treated with *L. plantarum* (Fig. 2A). This observation remained consistent when examining bone parameters of trabecular bone volume, trabecular thickness, trabecular number, trabecular separation, and cortical bone thickness (Fig. 2B-F). In agreement, *L. plantarum* treatment in normal mice did not induce any up- or down-regulation of trabecular or cortical bone in femurs compared to the PBS-treated mice, as evident in H&E staining results (Fig. 2G). Differentiated bone cells, both osteoclasts and osteoblasts, on the bone surface were not changed by the *L. plantarum* treatment (Fig. 2H-K). Collectively, intragastric administration of *L. plantarum* does not change the bone mass when administered to normal healthy mice.

### Intraperitoneal Administration of *L. plantarum* Induced Femoral Bone Destruction

**Fig. 3:**
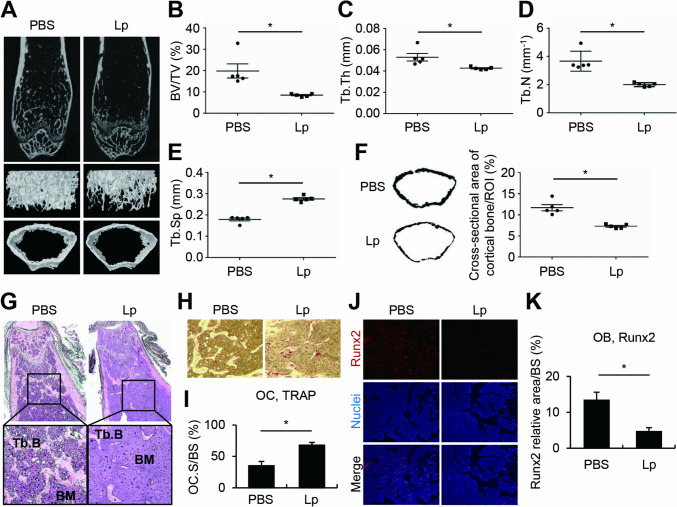
Intraperitoneal administration of *L. plantarum* augmented femoral bone destruction. C57BL/6 mice (*n* = 5) were intraperitoneally administered heat-treated *L. plantarum* (2 × 10^9^ CFU) for four weeks at a four-day interval. (**A**) The femurs were scanned by micro-CT and a representative image of the femur is shown. (B-F) Bone parameters of (**B**) BV/TV, (**C**) Tb.Th, (**D**) Tb.N, (**E**) Tb.Sp, and (**F**) cortical bone thickness were calculated from the 3D reconstruction of micro-CT images using a CT analyzer and ImageJ software. (G-K) Sections from the femur were stained for (**G**) H&E, (**H**, **I**) TRAP, and (J, K) Runx2. Lp, heat-treated *L. plantarum*; BV/TV, trabecular bone volume; Tb.Th, trabecular thickness; Tb.N, trabecular number; Tb.Sp, trabecular separation; ROI, region of interest; Tb.B, trabecular bone; BM, bone marrow; OC, osteoclast; OC.S/BS, osteoclast surface/bone surface; OB, osteoblast; BS, bone surface. *P < 0.05

To determine the effect of *L. plantarum* administered by a route other than intragastric, we administered *L. plantarum* via intraperitoneal injection to six-week-old healthy normal mice for four weeks at a four-day interval. Interestingly, unlike the results from intragastric administration, intraperitoneal injection of *L. plantarum* induced femoral bone destruction (Fig. 3A), with reduced trabecular bone volume, thickness, and number and augmented separation (Fig. 3B-E). *L. plantarum* treatment not only decreased trabecular bone mass, but also thinned cortical bones compared to those of PBS-treated mice (Fig. 3F). We also demonstrated that *L. plantarum* treatment reduced bone mass of normal mice with the upregulation of osteoclasts and the downregulation of osteoblasts through H&E, TRAP, or Runx2-specific immunofluorescent staining of femurs (Fig. 3G-K). These results suggest that, unlike intragastric administration, intraperitoneal injection of *L. plantarum* augmented bone destruction with increasing osteoclastogenesis and decreasing osteoblastogenesis.

### *L. plantarum* directly Upregulated Osteoclastogenesis and Downregulated Osteoblastogenesis

**Fig. 4:**
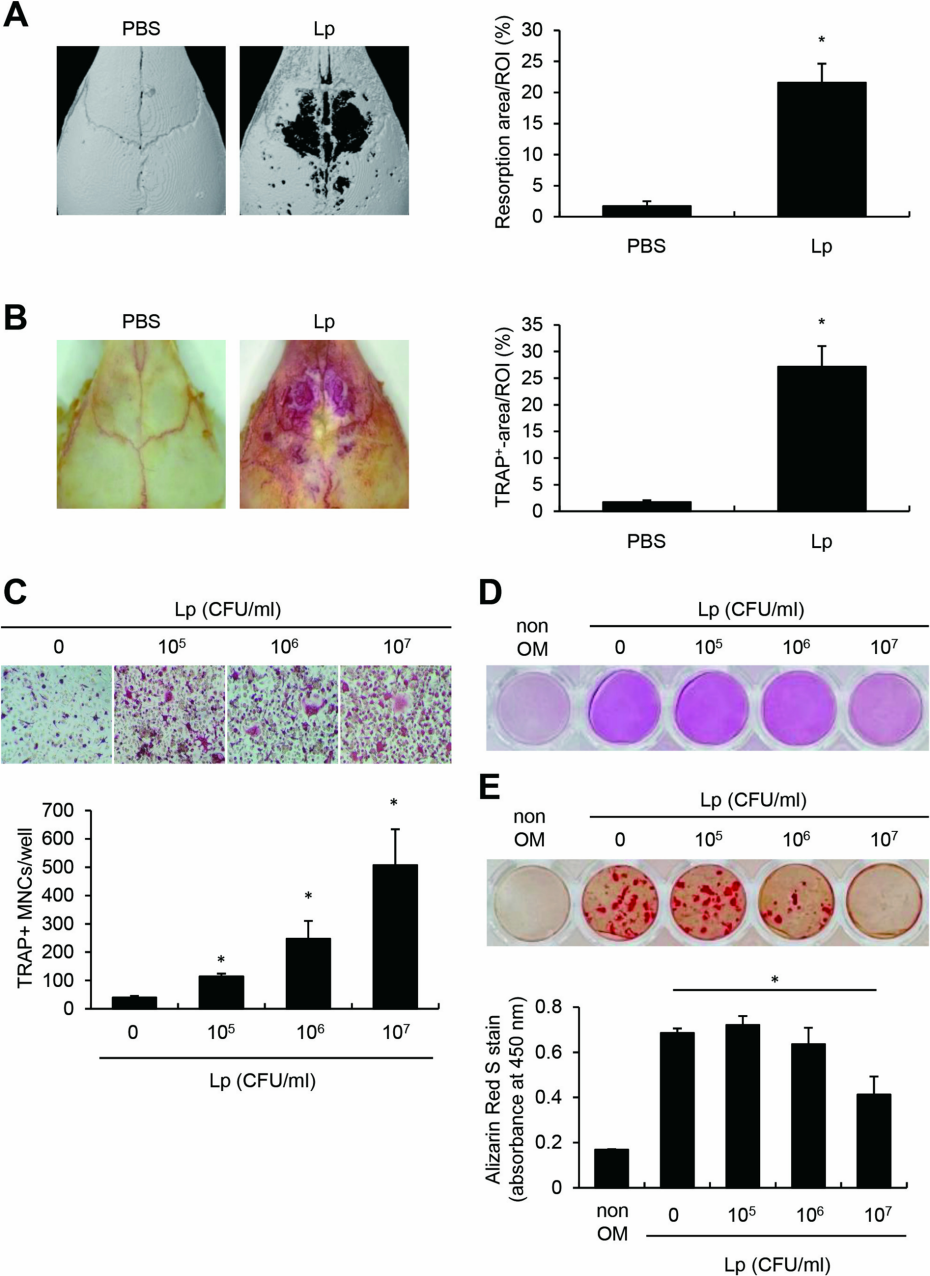
*L. plantarum* directly upregulated osteoclast differentiation and downregulated osteoblast differentiation. (A-B) A collagen sheet soaked with heat-treated *L. plantarum* (1 × 10^9^ CFU) was implanted on the calvaria of C57BL/6 mice (*n* = 5) for seven days. (**A**) The calvarial bone was scanned by micro-CT and a representative image is shown. The resorption area was analyzed by ImageJ software. (**B**) The calvaria was subjected to TRAP staining and photographed. The TRAP-stained area was analyzed by ImageJ software. (**C**) Committed osteoclasts were treated with M-CSF (30 ng/ml) and *L. plantarum* for 24 h. After incubation, they were fixed and subjected to TRAP staining. The cells with three or more nuclei were enumerated by microscopy. (**D**) MC3T3-E1 cells were treated with 2 mM β-glycerophosphate, 50 µg/ml ascorbic acid, and *L. plantarum* at a two-day interval for six days. The cells were fixed and stained with an ALP-staining kit. (**E**) Primary osteoblasts from calvaria of one-day mice were differentiated for 12 days. The cells were fixed and subjected to alizarin red S staining. Lp, heat-treated *L. plantarum*; ROI, region of interest. *P < 0.05

To further investigate what happens when *L. plantarum* comes into direct contact with bone cells, we performed the mouse calvarial implantation surgery with *L. plantarum *in vivo and osteoclast or osteoblast differentiation experiments in vitro. A collagen sheet soaked with PBS or *L. plantarum* was implanted on each mouse calvaria. After seven days, calvarial bones were analyzed by micro-CT. Interestingly, *L. plantarum* treatment potently increased bone resorption area of calvaria compared to the PBS control group (Fig. 4A). The TRAP-stained area of calvaria was also higher in the *L. plantarum*-treated mice than in the PBS-treated mice (Fig. 4B). Additionally, we confirmed the effect of *L. plantarum* on osteoclast or osteoblast differentiation in vitro. Concordant with Fig. 4A and B, L*. plantarum* directly augmented osteoclast differentiation (Fig. 4C) and decreased osteoblast differentiation in a dose-dependent manner (Fig. 4D and E). Taken together, direct contact with bone cells and *L. plantarum* increases osteoclast differentiation and decreases osteoblast differentiation, resulting in bone resorption.

## Discussion

Accumulating reports with various in vivo and in vitro studies demonstrated that probiotics such as *Lactobacillus* species are beneficial to bone health [7]. Nonetheless, unintended adverse effects on bone metabolism were also reported [19]. Thus, it might be important to clarify under what conditions probiotics are beneficial to bone health. We found that intragastric administration of *L. plantarum* to OVX-induced osteoporotic mice resulted in increased bone density. However, intriguingly, no such bone-enhancing effects were observed when *L. plantarum* was intragastrically administered to healthy normal mice. Additionally, bone volume was reduced when *L. plantarum* was administered intraperitoneally as opposed to intragastrically. In vitro experiments to validate these observations demonstrated that *L. plantarum* treatment led to an increase in osteoclastogenesis and a decrease in osteoblastogenesis. Concordantly, direct administration of *L. plantarum* on calvarial bone induced bone resorption in vivo. Our results showed that *L. plantarum* had distinct roles in the regulation of bone homeostasis depending on delivery routes and the status of bone health.

Intragastric administration of *L. plantarum* increased the femoral bone volume in OVX-induced osteoporotic mice, while no such effect was observed in healthy normal mice. These findings are consistent with previous research. Probiotics, recognized for their effectiveness in promoting bone healing, have shown positive outcomes in osteoporosis mouse model [27–30]. For instance, oral administration of *Lacticaseibacillus rhamnosus* to OVX mice results in a higher bone volume compared to OVX mice given PBS [27]. Similarly, OVX mice consuming water containing either *Lacticaseibacillus paracasei* alone or a combination of *L. paracasei* and *L. plantarum* exhibit higher bone mineral content than those without probiotic supplementation [28]. Conversely, probiotic treatments in sham control mice do not elicit changes in bone mass [28]. In a similar manner to our data, McCabe et al. demonstrated that intragastric administration of live *Limosilactobacillus reuteri* to healthy female mice does not alter the bone volume of the femur or vertebrae [31]. Considering our results and the others, oral intake of probiotics could potentially serve as an effective strategy for recovering bone health in patients with osteoporosis but not those with normal bone density.

The absence of bone-increasing effects in normal mice and the manifestation of bone-augmenting effects exclusively in osteoporotic mice by *L. plantarum* consumption might be attributed to two main factors; the alleviation of inflammation and the changes in microbiota composition. First, *L. plantarum* may recover femoral bone volume by controlling inflammatory responses in OVX mice. Estrogen deficiency leads to the high production of pro-inflammatory cytokines such as interleukin (IL)−1, IL-6, IL-17, tumor necrosis factor-α, and interferon-γ and eventually results in bone loss [31–34]. Considering the effect of *L. plantarum* in reducing cytokine levels [35], the benefits might not be applicable to normal mice without inflammation. In this context, the primary mechanism for increasing bone could involve reducing inflammation in osteoporotic mice. Second, oral intake of *L. plantarum* might promote the recovery of OVX-induced dysbiosis of gut microbiota, leading to increased bone density. For instance, *L. rhamnosus* alters the gastrointestinal microbiome in OVX rats and results in the amelioration of OVX-induced osteoporosis [36]. The consumption of *L. rhamnosus* induces bone formation by increasing the relative abundance of *Firmicutes* and *Desulfobacterota*, while decreasing *Bacteroidetes* in an OVX group [36]. Similarly, clinical data indicated that patients with osteoporosis undergo alterations in gut environment and microbial composition [37–39]. The consumption of probiotics helps these osteoporotic patients establish a healthy normal microbial flora, promoting gut microbial homeostasis and ultimately enhancing bone health [6, 40]. However, further studies are anticipated to verify the exact mechanisms underlying the recovery of bone mass by *L. plantarum* in OVX mice.

Intraperitoneal injection or calvarial implantation of *L. plantarum* exacerbated bone destruction, contrasting with the results observed in intragastric injection. This observation suggested that the translocation of *L. plantarum* from the intestine to other parts of the body gave rise to potential problems. Consistent with this, in certain clinical cases, *Lactobacillus* species are implicated as opportunistic pathogens causing bacteremia, dental abscesses, and prosthetic knee infections [41–43]. The adverse effects of probiotics outside the intestines might be attributed to the presence of bacterial lipoproteins. When probiotics enter the gastrointestinal tract, they undergo digestion, resulting in the release of effective molecules such as peptidoglycans and their fragmented small building blocks [40, 44–46]. Conversely, when probiotics enter through alternative routes, they may remain undigested, persist as whole bacteria, and act as opportunistic pathogens with their lipoproteins as major virulence factors. Indeed, bacterial lipoproteins are the strongest immune stimulators [47, 48] and are a major source of bone destruction, particularly in Gram-positive bacterial infections [49]. Undigested *L. plantarum* containing lipoproteins may strongly activate the Toll-like receptor 2 signaling pathway [50, 51], leading to the upregulation of osteoclast differentiation and the downregulation of osteoblast differentiation. In summary, undigested *L. plantarum* that infects areas outside the intestines functions as opportunistic pathogens, potentially causing bone destruction through the action of their lipoproteins. In addition, prebiotics, which promote the growth of probiotics by serving as their nutrients, and synbiotics, a combination of prebiotics and probiotics, have been shown to improve bone health [52, 53]. However, it remains unclear whether the effects of prebiotics and synbiotics on bone health vary according to delivery route. Therefore, further studies are needed to clarify whether the delivery routes of prebiotics and synbiotics impact their effects on bone health.

Probiotics are widely recognized for their health benefits, including the prevention of antibiotic-associated diarrhea, treatment of periodontal disease, and alleviation of ulcerative colitis [54]. However, probiotics could be harmful to people with compromised immune systems or health issues [55, 56] by eliciting the adverse effects such as systemic or local infections and allergic reactions [57]. In this study, we demonstrated that probiotics can be either beneficial or harmful, depending on the host’s health conditions and the routes of administration. These experimental conditions are likely critical for determining the optimal probiotic strains, dosages, and administration routes tailored to a specific host’s health status. To maximize the benefits of probiotics and minimize the adverse effects, further studies are needed to identify beneficial probiotic strains and clearly understand the mechanisms for their pleiotropic actions underlying the interaction between probiotics and host.

In conclusion, the intragastric administration of *L. plantarum* was highly effective in treating osteoporotic bones but had no effects on normal healthy bones. On the other hand, when *L. plantarum* was introduced through a route other than the intestine, it diminished bone mass. The effects of probiotics are not universally positive and can vary significantly depending on the bacterial strain. Additionally, delivery routes of probiotics can influence its efficacy. Therefore, understanding how different delivery routes impact probiotic effectiveness is essential. However, no studies to date have investigated the efficacy of probiotics across various delivery routes. Our study clearly demonstrates that both individual health status and the route of probiotic administration are crucial factors to consider when consuming probiotics.

## Data Availability

No datasets were generated or analysed during the current study.
